# Mathematically Gifted Students’ Experience With Their Teachers’ Mathematical Competence and Boredom in School: A Qualitative Interview Study

**DOI:** 10.3389/fpsyg.2022.876350

**Published:** 2022-05-30

**Authors:** Jørgen Hammer Smedsrud, Anders Nordahl-Hansen, Ella Idsøe

**Affiliations:** ^1^Nordic Institute for Studies in Innovation, Research and Education, Oslo, Norway; ^2^Department of Education, ICT and Learning, Østfold University College, Halden, Norway; ^3^Norwegian Centre for Science Education, University of Oslo, Oslo, Norway

**Keywords:** mathematically gifted, gifted students, teaching competence, mathematical creativity, boredom

## Abstract

Competent mathematics teachers who have knowledge of gifted students’ needs can challenge them in math and prevent boredom and possible underachievement. This retrospective study explores how Norwegian gifted students perceive their earlier teachers’ mathematical competency, as well as their reflections about boredom in school. The data were collected through qualitative semistructured interviews with 11 mathematically gifted students who participated in accelerated classes throughout school. The informants ranged in age from 16 to 19 years and were asked about how they experienced their math classes, teachers, and social aspects. The results indicate that students view their teachers as having less mathematical knowledge in earlier school than in later years and that teachers’ mathematical knowledge might affect whether they are able to challenge and identify students who are gifted in mathematics.

## Introduction

In Norway, there are few to no resources for mathematically gifted students in ordinary schooling outside skipping a grade (acceleration). However, we know little about how the teachers approach this group in both ordinary schooling and accelerated classes. Therefore, we need knowledge about how mathematically gifted students experience school and how teachers can meet their needs. High-quality instructional practices lead to dedicated students and cognitive activation (Blömeke et al., 2016). Student learning outcomes are determined by high-quality teachers with high levels of subject knowledge as well as the ability to unite knowledge with practice. Although researchers have emphasized the relationship between instructional practice and teachers’ mathematical knowledge concerning student improvement in mathematics, they struggle to quantify the items meant to measure these abilities (Nilsen et al., 2016). The latter describe two levels of how mathematical knowledge is understood: through *intellectual* and *policy* views. Policy views here refer to politics or ideologies guiding, for example, teacher education, education in mathematics, and/or the curriculum. In the intellectual view, mathematical knowledge consists of comprehension of mathematical methods, facts, rules, terms and concepts and procedural skills such as rule-based operations (Niss et al., 2016). To score highly in these categories, the student needs to apply a wide range of cognitive skills (Grønmo et al., 2013). Mesa et al. (2013) suggest that, in Europe, the content of the curriculum is traditionally more the focus rather than teachers’ ability to instruct students. This tradition is reflected in the learning goals provided through policies. The learning goals reflect standardized goals for a given age group in school (Mesa et al., 2013). Furthermore, the categories represented by the *Teacher Education and Development Study in Mathematics* (TEDS-M) tend to garner more attention than a possible relationship among instructional quality, achievement and motivation. TEDS-M consists of three overarching categories, with the aim of describing the quality of teacher education as follows: (1) organization, where the goal is to describe how teacher education is organized and policies related to education; (2) the quality of local teacher education and how it is organized; and (3) the teachers’ students’ knowledge of mathematics, math instruction, and mathematical didactical knowledge (Grønmo and Onstad, 2012). As an explanation, the teacher’s knowledge of mathematics (instruction and knowledge) seems to receive more attention than the student’s knowledge, motivation, and quality of instruction. At the same time, school climate and instructional quality also seem to affect individual motivation in mathematics (Scherer and Nilsen, 2016). Defining mathematical literacy through the curriculum can be troublesome because mathematically gifted students process and/or perceive mathematics in alternative ways for which the curriculum allows (Assouline and Lupkowski-Shoplik, 2011; Leikin et al., 2017).

### Mathematically Gifted Students and Their Needs

There are a variety of general theoretical models of giftedness that could include a framework for explaining mathematical giftedness (Leikin, 2021). Among these models, we mention Gagné’s Differentiated Model of Giftedness (Gagné, 2005), Renzulli’s tripartite model (Renzulli, 1988), and Ziegler‘s Actiotope Model of Giftedness (Ziegler, 2005). In line with some researchers in mathematical giftedness (e.g., Leikin, 2011; Szabo, 2015) and for the purpose of our study, we will focus on explaining the characteristics of mathematically gifted individuals from a conceptual perspective. The domain specificity of mathematical giftedness always implies a collection of certain mathematical abilities and personal qualities. Generally, students who are gifted in mathematics are described as students with strong *problem-solving abilities, metacognitive abilities, creative mathematical thinking, and high ability/performance in mathematical problem solving* (Leikin, 2014; Leikin et al., 2017). Mathematical giftedness is also seen as an inherent potential for mathematical knowledge and a deeper understanding of mathematical concepts (Leikin et al., 2009; Subotnik et al., 2009). Mathematically gifted individuals possess intellectual characteristics, such as curiosity, the ability to visualize models, quick thinking and metaphorical thinking (Silverman, 1997; Deary, 2000). Mathematical creativity is also mentioned as a characteristic among these students even though there is no commonly accepted definition of the term (Plucker et al., 2004a; Singer et al., 2017). To capture different ways of being creative in mathematics, some recent studies take a different approach to creativity and adopt the concept of cognitive flexibility, which is explained as an interplay among cognitive variety, cognitive novelty, and changes in cognitive framing (Pelczer et al., 2013; Voica and Singer, 2013; Zhang et al., 2017; Schoevers et al., 2020). Mathematical creativity also seems to promote self-efficacy (Bicer et al., 2020; Regier and Savic, 2020). Studies show that intellectual test scores should supplement dynamic and informal data, such as information from parents, teachers, or other students (Al-Hroub, 2011). These studies might have important implications for both identifying giftedness in mathematics and for nurturing such students’ needs.

Empirical evidence suggests that the main element in fostering mathematically gifted students is learning opportunities (Nadjafikhah et al., 2012; Hoth et al., 2017). Studies have suggested that teachers lack the knowledge to provide gifted students with the appropriate cognitive challenges in the regular classroom (Diezmann, 2005; Hoth et al., 2017). Instead, their instruction typically focuses on tasks aimed at the general student population (Rotigel and Fello, 2004). Steenbergen-Hu et al. (2020) found that underachieving gifted students might score lower in aspects connected to self-regulation and motivation. The latter might indicate that they have a lower “threshold” to become bored and thus need to be challenged to maintain their motivation. Barbier et al. (2022) found that enhancing self-regulated learning and providing differentiated instruction might affect gifted students’ motivation. A learning environment that can meet the needs of students gifted in mathematics is recognized by an appreciation for alternative ideas and discussion of alternative or multiple solutions (Nadjafikhah et al., 2012; Ronksley-Pavia and Neumann, 2020). Teachers should provide guidance for students to explore their own ideas, define and make hypotheses, refute and adapt heuristic strategies, and reason and justify conclusions and reflect on them at a metacognitive level (Nadjafikhah et al., 2012; Hoth et al., 2017).

### Teachers’ Mathematical Competence in Teaching Gifted Students

In the literature, three core dimensions of teachers’ mathematical understanding are important: *content knowledge* (MCK), *pedagogical content knowledge* (MPCK), and generic *pedagogical knowledge* (GCK) (Baumert et al., 2010). TEDS-M and the follow-up study, TEDS-FU, hypothesize two dimensions, MCK and MPCK. The TEDS-M framework was developed through extensive research on the international pool of items measuring mathematical knowledge (Kaiser et al., 2017). There are associations between these dimensions and the ability to communicate mathematics in the classroom, as well as the teacher’s ability to deconstruct knowledge and support both high and low achievers (Ball et al., 2001; Baumert et al., 2010). TEDS-M MCK includes the main mathematical areas relevant for future teachers. MPCK includes knowledge for lesson planning and knowledge applied to teaching situations. Finally, GPK includes knowledge about teaching, learning, and evaluating student achievement (Kaiser et al., 2017). Studies have suggested relationships among the categories, where MPCK needs to be accompanied by mathematical knowledge and skills related to curriculum understanding to be an effective teaching tool (Baumert et al., 2010). In Hill et al.’s (2005) study, mathematical knowledge for teaching included explaining terms and concepts to students; interpreting student statements and solutions; judging and correcting textbook treatments of topics; using representations accurately; and providing students with examples of mathematical concepts, algorithms, or proofs. The authors found that teachers’ mathematical knowledge positively predicts student improvement in mathematics (Hill et al., 2005). Teachers’ mathematical content knowledge MCK and pedagogical content knowledge MPCK are portrayed as having an important relationship with student gains in mathematical knowledge across levels of performance (Baumert et al., 2010), indicating that focusing on mathematical knowledge would be advantageous for all students. At the same time, some studies suggest that the joint effect of classroom management has a stronger effect on mathematical achievement than individual support (Baumert et al., 2010). In Baumert et al. (2010), MCK only affects teachers’ ability to adjust the material covered in the curriculum for weaker students. Research suggests that adjusting the material through acceleration is one of the best ways of meeting gifted students’ needs (Colangelo et al., 2004), specifically for those who are high achievers (Colangelo and Assouline, 2009). Gifted students need cognitive challenges to feel accepted and develop positively in school (Kulik and Kulik, 1992). The opposite of challenge is boredom, which might be a way of coping with a lack of challenge.

### Boredom as Lack of Challenge

Studies indicate that gifted students have higher intrinsic motivation than other students (Gottfried et al., 2005). Moreover, it is suggested that motivation determines the difference between high- and low-achieving gifted students (Reis and McCoach, 2000; McCoach and Siegle, 2003b). Boredom is often mentioned in relation to gifted students in school or as an argument for providing gifted students with opportunities in school (Preckel et al., 2010). Boredom is commonly understood as an affective state that comprises weak feelings, lack of stimulation and low psychological arousal; implies more than the absence of positive emotions and interests; and is a subjective experience (Preckel et al., 2010). Moreover, studies have suggested that boredom might contribute to underachievement among gifted students (Reis and McCoach, 2000). In particular, boredom with the regular curriculum in elementary and middle school contributes to underachievement in high school (Baker et al., 1998; Reis and McCoach, 2000; Kanevsky and Keighley, 2003). Studies focusing on boredom as a result of little challenge have received more attention than studies that relate boredom to overchallenge (Preckel et al., 2010). In academic settings, it seems that students differentiate between task-focus and self-focus boredom (Acee et al., 2010). Students seem to relate boredom from overchallenge to two dimensions, namely, situation and task focus-related boredom, whereas boredom due to underchallenge does not seem to relate to a general boredom factor. Moreover, task-focused boredom can be characterized by students’ focus on the tediousness and meaninglessness of the task, whereas self-focused boredom can be characterized by students’ focus on their feelings of dissatisfaction and/or frustration (Acee et al., 2010). However, single studies seem to imply that gifted students experience boredom more frequently than non-gifted students (Plucker et al., 2004b). A study by Preckel et al. (2010) indicated that gifted students experience more boredom due to underchallenge, and non-gifted students experience boredom due to overchallenge; at the same time, this study does not seem to imply that gifted students experience more boredom than other groups. A study by Feuchter and Preckel (2021) indicated that ability grouping for gifted students might have positive academic outcomes and does not seem to prevent boredom among the same group. Thus, compensation for boredom might be more suitable for the regular classroom. One way that teachers could prevent boredom among mathematically gifted students is to design tasks that present an individually optimal level of stimulation, but this requires profound knowledge of students’ ability level and learning preferences (Westgate and Wilson, 2018).

## The Present Study

A qualitative study by Smedsrud (2018) explored how students gifted in mathematics experienced participation in accelerated and high-ability groups. The results indicate that the students reaped academic benefits from acceleration, and they did not describe any social and/or emotional harm from participating in these programs. In the current study, we further investigate their descriptions of their mathematics teachers during school and whether they were challenged in math during their ordinary classes. The open nature of the interviews enables further exploring them and connecting them to other subjects in gifted education and teacher education, particularly if the competence among teachers in Norway makes it beneficial to facilitate learning opportunities in accelerated and high-ability groups.

A first and major goal of the present study was to investigate how gifted students experienced their teachers’ mathematical competence. The second goal was to explore how gifted students coped with boredom throughout school. The research questions for this study are as follows:

1. How do students experience their teachers’ mathematical content knowledge?
2. How do students experience their teachers’ mathematical pedagogical knowledge?
3. How do students cope with boredom throughout school?

## Research Design

### Methods

The present study draws on qualitative thematic analysis (Braun and Clarke, 2006) with data collected from semistructured interviews with 11 mathematically gifted students who participated in acceleration opportunities throughout Norwegian school. The study topics were selected based on the information given by the students in the interviews. We developed a semistructured interview guide based on exploring students’ school history, whether they experienced challenges in ordinary school, and how the acceleration program differed from their ordinary classroom instruction and was connected to motivation in mathematics and/or other school subjects. The open nature of the interviews enables further exploring them and connecting them to other subjects in gifted education and teacher education. For example, we did not directly ask the students about their teachers’ mathematical competency; however, all the students seemed to bring this theme forward as an important factor influencing whether they were challenged in their ordinary classes. Although the original interviews were in Norwegian, we have translated some examples of questions that were addressed, such as the following: *How would you describe mathematics as a subject in school? Can you describe your school experience in general and specifically regarding mathematics? If you could decide, how would the teacher teach mathematics in school?*

#### Informants

The participants in this study were 11 students, three girls and eight boys (age range from 16 to 19). These students participated in a mathematics *high-ability group* at a Norwegian higher education institution and had received *acceleration* opportunities throughout school. The participants were selected to the acceleration group by their math scores in grade five or six^1^ and by application to participate. All the participants came from public schooling and had a wide range of socioeconomic backgrounds, and the only criterion to participate was high grades and thus a performance-based nomination. The selection of participants is a preselected convenience sample (Gorard, 2001) that shares one common denominator, which is participation in the acceleration program at the university.

#### Procedure

The Norwegian Center for Research Data approved this study. The consent obtained from all the participants was both written and informed. One participant withdrew from the study. As all the other information from the study was deleted in line with NSD guidelines, we had no information other than sex. The first author performed individual semistructured interviews with the 11 gifted students. The interviews lasted from 1 h to one and a half hours in some cases. The students were asked follow-up questions whenever something was unclear or the informants touched on something that seemed interesting.

#### Analysis

The interviews were transcribed in Microsoft Word and analyzed a second time in NVivo version 11™ (Castleberry, 2012). We used thematic analysis as an analytical method for our qualitative study. Thematic analysis is the most widely used qualitative approach to analyzing interviews (Braun et al., 2019). According to Braun and Clarke (2006, p. 79), thematic analysis is a method used for “identifying, analyzing, and reporting patterns (themes) within the data.” In the present study, we explored the data further by asking new questions about them and making different interpretations from the original research and repurposing with other research question(s). The latter allowed us to identify the following main categories: 1. *Teachers’ mathematical content knowledge*, 2. *Teachers’ mathematical pedagogical content knowledge*, 3. *Students coping with boredom*, and 4. *Possible consequences of boredom for gifted students.* The analysis in use is inductive thematic analysis, which means that some of the categories discussed in this paper are developed from the data and the overall themes are reflected in the interview guide.

## Results

The main research question in our study is related to gifted students’ experience of their mathematics teachers’ competence. As we present the results and discussion in this article, it is important to acknowledge that we have no information from the specific teachers in this study. Therefore, the teachers’ mathematical competence is described retrospectively from the students’ perspective and therefore is based on that of any teachers they have had rather than a specific one. Although we do not have a teacher’s perspective, students’ voices are very important in improving education and promoting engagement in school (Mitra, 2005; Cook-Sather, 2007).

### Students’ Experience With Teachers’ Mathematical Content Knowledge

As mentioned earlier, mathematical content knowledge is associated with teachers’ ability to understand mathematics and influences their ability to communicate and support both high- and low-achieving students. In the interviews, several students addressed the importance of knowledge among teachers in communicating with and challenging them in mathematics. In some cases, they had had teachers who had this ability in senior high school, but few had experienced it in earlier schooling. The student below described the lack of challenges that she received during primary school and how the teacher chose to use her as a resource in the classroom instead of giving her more challenges in mathematics: “In primary school, if you finished your work early you should receive a new challenge with variations, at least be allowed to work somewhere else. Instead, I had to help other students who were not finished yet” (Girl).

In contrast to recommendations for teachers, the student was used as a teacher assistant in class. This could have affected the social climate, and the student might have felt outside the normal group in the classroom. Furthermore, the reason she did not receive any challenges in math could be that the teacher lacked the proper content knowledge to present any. For example, most of the students received “more” of the same mathematical problems that they had already solved rather than new challenges or creative assignments. As one informant stated, “*In the lower end school, you receive a problem and are presented with one answer, as if that is the only answer or solution. However, a good student can find several answers to some mathematical problems*” (Boy).

As described earlier, teachers with low mathematical content knowledge would not be able to recognize creative answers or strategies for solving a mathematical problem other than a “*standard.*” Additionally, they might be able to give students more assignments but not necessarily enrich the subject or use differentiated strategies in mathematics. One student described the teachers’ attempt at differentiation as follows:

Sometimes I received a sheet with a lot of much harder mathematical problems, it was ok. However, the teacher did not support me or tutor me in the subjects, so it became too hard. I did not get that much out of those challenges (Boy).

Here, the teacher seems to have found some mathematical problems that were challenging for the student. However, many gifted students still need support and/or guidance in working on such assignments. The reason the teacher did not support the student could be because he or she lacked high-level insight and mathematical content knowledge and, thus, could not provide the student any more guidance than adjusting the curriculum or simply lacked time to prepare, differentiate among and teach the different students in class. If the teacher must teach mathematically gifted students at their levels of understanding, they need high mathematical content knowledge. “*Effective teachers are perceived as those who know how to make the triadic relationship between content or subject matter, pedagogy or teaching strategies, and the student population*” (Kaplan, 2003, p. 1). A teacher’s ability to transform content knowledge into pedagogical strategies can be essential to meet mathematically gifted students’ needs so that the students do not become bored in school, increasing their risk of becoming underachievers.

### Student Experience With Teachers’ Mathematical Pedagogical Content Knowledge

As Thompson (1984) noted, teachers’ beliefs about, views of, and preferences in mathematics play an important and significant role in shaping their instructional behavior. Teachers’ mathematical knowledge can, in this way, affect their interest and ability to communicate mathematics to students and, thus, play an important role in how they can meet mathematically gifted students’ needs. One of the informants in the study summarized how he experienced his teachers’ ability to teach mathematics in the following way:

For the average secondary school teacher, it seems like they barely know what they are going to teach the students. They can explain what is relevant for that year and maybe 1 year over what they are supposed to teach (Boy).

Another student described how he liked to use mathematics to solve real-life problems by calculating the *dimension of tubes* or the *weight of a building* and felt that most teachers lacked the relevant knowledge to guide him in these interests. Although we cannot expect most teachers to have the mathematical knowledge of engineers or math professors, they should recognize the need for differentiation and support for the students at the top end of mathematical knowledge. One of the informants described the need for challenge and support: “*For example, me and my friend could work with more difficult calculation in math. That is a good thing, however, the teacher must teach us the concepts and support us in the process*” (Boy).

In this study, the typical way that the students’ needs were acknowledged was through exclusion. The students with higher mathematical ability often received some opportunities before acceleration and thus little support or guidance:

Yes, sometimes I asked the teacher because I wanted to work with something else. It was sometimes ok that we went out in the hallway, so we did not disrupt the rest of the class. However, I have also been told by the teacher to sit at the back of the classroom, so I do not disturb anyone else (Girl).

This tendency was described as greater at the lower end of schooling (primary and secondary school) than in senior high school. However, the students also stated that they generally did not need the teacher to understand mathematics, indicating that they were not challenged beyond their understanding of the subject.

In 9th (junior high school), the teacher allowed me and some friends to work with the material for 10th grade, which we found ourselves. It was in that sense “ok” to be good in the subject. We could sit in a corner and work us three together (Boy).

The lack of challenges in mathematics affected the students in different ways. It seemed that acceleration only gave them some challenges, particularly when the volume of training in mathematics was connected to grade skipping. However, in-depth enrichment and instruction seemed to be lacking until they participated in a group at the university. For example, one student described how she suddenly needed support from the teacher to understand the assignments:

Earlier, the challenges were a little here and there. This year, it has been good and challenging. The subjects we had at the university were very good and challenging. It was different, with different tasks, more thinking. Not like earlier where the teacher gave you a calculation and then gave the answer. I feel like I needed the teacher at the university, much more than earlier in school (Girl).

The fact that the student needed teacher support to perform at the university indicated that she was challenged within her proximal zone of development. At the university, the teacher had a different approach to the subject and, at the same time, greater mathematical knowledge. Moreover, the teacher had students who were interested in the subjects. It is interesting that several students expressed that they did not need academic support from teachers until they took courses at the university, where the level of instruction was very high. Some students did not need the teacher at the university either and seemed to rely on personal preference. Some students liked to work on a problem over time, while others liked for the teacher to help and guide them. For many, the content knowledge and pedagogical content knowledge of the teacher at the university were beyond expectations. Those students experienced the group at the university as completely different in a positive way. Interestingly, the students who wanted support and the students who liked to work alone experienced the university group as positive, indicating the teacher’s ability to differentiate among both subjects and individual preferences.

### How Students Cope With Boredom Throughout School

All the informants described their motivation as varying throughout school, which is normal and expected for all students. Their motivation depended on the teacher who taught the subject and the teacher’s level of knowledge in that subject. Boredom seemed to dominate their school years across all cases, especially in the earlier years. Although we focus on mathematics in this article, the students felt bored across all subjects of interest. One student described the feeling of boredom as follows:

In science, there was much focus on the weaker students. There was much repetition, it seems they wanted more students in the third and fourth grades (middle grades in Norway), instead of sixth (highest grade). I was bored all the time in the science classes in secondary school (Boy).

It is unclear whether the lack of challenge in this case was connected to teachers’ knowledge within the specific subject or to how the teachers initially focused on the average students in class and, therefore, missed opportunities to differentiate for gifted students often described as “*scaffolding.*” The informants connected boredom to *repetition*, *pace*, and *depth*, especially in mathematics: “*At least in eighth and ninth grades, it was repetition. I was very bored, and it became even more boring”* (Boy). As mentioned, it seems that the informants were more bored in their regular classes during middle school than earlier and at later points in their education. At the same time, many received their first opportunities to accelerate or skip grades during this time. It could also be that their ability to reflect on their own situation improved in middle school from earlier years and that they also recognized that they were bored because they had experienced more motivating settings. As one student reflected regarding his schooling, “*It was very boring. Now, subsequently, I realize that it probably was because it was very easy, but I did not reflect over that at that time. I just remember that math was not exciting anymore”* (Boy).

### Possible Consequences of Boredom for Gifted Students

The lack of individual understanding and experience of a meaningful education had different consequences for the students. Some felt they did not develop any good learning strategies. Others described how they at times developed what teachers might characterize as challenging behavior:

In primary school, I felt school was very boring, I did not take any of the subjects seriously back then. I had some energy and was a little hard to handle and was at the principal’s office many times, and I was different from the rest of the class, at least that was what I felt (Boy).

Other students became passive and did not truly engage in classroom activities. Instead, they only participated if they were directly asked questions. There was a difference between experiencing boredom over time and being bored in some situations or moments during school, and this difference seems to be important for whether the students developed difficult relationships with teachers or the school. Several informants noted that there were other gifted students with whom they had participated in accelerated classes or whom they knew who did not continue in those classes and became troublemakers or lost interest in the subject:

I think many smart and gifted children/students who are smart and gifted when they are young become troublemakers and disrupt the classroom situation because they are not challenged. And therefore, the teachers do not think they are smart because they do not do or produce anything in class; they only think they are troublemakers. Therefore, I think it is very important to give them differentiated education or at least find out if they are smart (Girl).

Interestingly, the students described how they experienced other students becoming apathetic or dropping out of similar programs. It could be that the personality of the individual student is important in determining whether they underachieve in school. As the informant noted, it could be that some gifted students become troublemakers early in school and, therefore, are not even recognized as potential high achievers. As mentioned previously, teachers need high mathematical competence to both challenge and identify mathematically gifted students. The informants’ statements indicate that it is merely by chance whether students are challenged and, thus, identified as gifted or high achievers.

## Discussion

The main purpose of this study was to investigate how students experience their teachers’ mathematical content knowledge. How do students experience their teachers’ mathematical pedagogical knowledge? How do students cope with boredom throughout school?

The informants in this study noted that although they received few differentiated assignments, they received *more of the same* or, in some cases, experienced increased difficulty in their regular schooling. There seems to be a connection between primary teachers’ MCK and their ability to support and identify creative and high-achieving students (Hoth et al., 2017). The current study indicates that only teachers at competence level three (TED-M)^2^ could meet the needs of high-ability students during class. Moreover, the same tendencies were displayed between MPCK and teachers’ ability to support creative and high-achieving students during class. At lower competence levels, teachers generally cannot support high-achieving students (Hoth et al., 2017). As a result, in Norway, there is insufficient ability to meet the needs of mathematically gifted students in regular classrooms. The findings in this study are also in line with those of Brevik et al. (2018). They interviewed teaching students at a central institution in Norway, and the teaching students stated that they had difficulty giving differentiated instructions to this student group in practice and responding to gifted students’ instructional needs. The creative aspect of mathematics was mentioned by the students in this study; however, few of them felt that there was room to express their creativity in class.

### Relationship Between Teacher Competence and Gifted Learners’ Needs

In classrooms where teachers cannot meet the needs of gifted students, acceleration might be one way to meet some of their educational needs. However, accelerating students does not necessarily serve as a way to address all aspects of their capacity for learning or their individual learning styles or personalities. A combination of enrichment activities and acceleration is often recommended for gifted students (Assouline and Lupkowski-Shoplik, 2011). Furthermore, there is no guarantee that teachers in accelerated classrooms have higher competency for teaching mathematics, which, in this case, is necessary for enrichment activities to be stimulating (Assouline and Lupkowski-Shoplik, 2011). A common misconception about gifted students is that they do not need any support or guidance in school (Montgomery, 2009). Nevertheless, in Norway, acceleration is only possible by skipping whole school years or placement in classrooms for older students. For a student to succeed in either case, he or she must display high levels of task commitment or else will be at risk of failing. The reason that only these types of acceleration are available to gifted students in Norway might be that Norwegian teachers are generally not comfortable with teaching high levels of mathematics in their own classroom; therefore, grade skipping is often used.

Teachers’ mathematical knowledge is important; however, some studies have suggested that a teacher can use strategies to stimulate gifted students without possessing the same level of knowledge (Assouline and Lupkowski-Shoplik, 2011). These strategies are aimed at stimulating problem-solving abilities. They involve open-ended tasks connected to practical settings. For example, students can discuss the pros and cons of the Fahrenheit vs. Celsius scales and why they are different. Although the teacher or student cannot provide a correct answer to this question, it can stimulate great learning opportunities in the classroom. Although teaching mathematically gifted learners may seem challenging, all teachers should be able to provide some opportunities. First, teachers need to train themselves to abandon the “lock-step” understanding of the math curriculum. Second, a curious teacher who is engaged in the subject can motivate all types of students. There is a large difference between dismissing a creative question because it may seem irrelevant and promoting curiosity by asking the student to explain her or his point of view. The latter is important for including students and can promote interesting learning opportunities in class. Teachers’ mathematical content knowledge matters; however, content expertise is not the entire story for effective pedagogy, and neither is an exclusive focus on instructional practice. The key to motivating all types of students is being an effective role model who demonstrates a passion for learning that is translated into action (Assouline and Lupkowski-Shoplik, 2011). Teachers need to assign more open-ended tasks and be sensitive to different (enriching) ways of understanding mathematics, even at lower levels of schooling.

### Students’ Thoughts About Boredom and Later Achievement

We herein discussed whether the ability to cope with boredom in school and experience later motivation seem to be important to how the students managed to cope with their daily learning situation. Boredom can be the result of having to wait to learn something or being far ahead of one’s peers (Kanevsky and Keighley, 2003). Although gifted students often have the individual capabilities to perform at a high level in school, they need to be motivated in the subject; if not, they might become unmotivated or underachievers (Gottfried et al., 2005; Phillips and Lindsay, 2006). Loss of motivation in gifted students can be connected to boredom (Little, 2012), which, again, can be connected to underachievement (McCoach and Siegle, 2003a) or the risk thereof (Reis and McCoach, 2000). Among the students in the present sample, many had experienced extensive boredom in school, and some even displayed problematic behavior. However, this did not seem to influence their later academic achievement, which is somewhat surprising. First, the students in this study seemed to conceptualize boredom as something connected to academic challenges rather than individual or social differences. Second, the students noted that they experienced boredom both as they participated in their classes and when they thought about their earlier school years, indicating that they were able to cope with situational boredom as it occurred, sometimes by concentrating on topics other than what was taught. A study by Adams-Byers et al. (2004) indicated that gifted students tend to see boredom as an academic issue rather than as connected to their social sphere. In the study, gifted students connected boredom to a slow pace and repetition of content, thus resulting in boredom. Repetition as a cause of boredom was also described in Pekrun et al. (2010). The latter also related the lack of research on boredom to the fact that boredom is a “silent” emotion; i.e., it is easier to access feelings, such as anger or anxiety, than boredom. An interesting comparison is in Brevik et al. (2018, p. 8), where teachers noted that one of the reasons that it is difficult to differentiate gifted students is that “.*they do as they are told.*” “*pay attention*,” and “*are interested*,” and several informants in this study expressed boredom in this “silent” way; thus, their teachers may not have realized that the students were actually bored. One of the informants displayed more problematic behavior, which resulted in several hours spent in the principal’s office. Therefore, frustration should also be understood as a possible indication of boredom or lack of motivation among gifted students (Kanevsky and Keighley, 2003). Boredom can promote motivation to avoid academic math and instead stimulate interests outside of the school setting (Pekrun et al., 2010). Additionally, a high *locus of control* and successful *boredom coping* are positively correlated (Goetz et al., 2006). By the same token, a central part of Renzulli’s three-ring model is *task commitment*. It might be that these students display high levels of task commitment and locus of control in that they, over time, are not affected by boredom in the same way that other student groups might be. Therefore, we also suggest that there might be a difference between boredom due to few challenges in school and boredom due to low mastery of the school subject. Initially, there might also be personality differences explaining why some students react with frustration and others by becoming apathetic.

## Conclusion

Research on MCK and MPCK emphasizes the relationship between a teacher’s mathematical knowledge and ability to communicate that knowledge to students. Furthermore, teachers with high scores in MCK and MPCK seem to be better at identifying and teaching gifted students in mathematics. In general, a teacher who possesses a high level of mathematical knowledge is better at organizing the material, knowing the rules, recognizing patterns, changing the representation of problems, guiding students in complex structures and working within these structures. Furthermore, these teachers recognize creative answers and are aware of multiple solutions. These features are necessary to meet the academic needs of mathematically gifted students. However, few of these studies have connected MCK and MPCK to high-ability or gifted students. Therefore, we have little knowledge about whether the needs of gifted students are met through acceleration or whether teachers who teach accelerated courses score higher on MCK or MPCK than the average teacher. In addition to including specific knowledge about the needs of gifted students in mathematics and evidence-based strategies to respond to these needs, a general increase in quality in the initial and continuing education of mathematics teachers could be very significant in making improvements. A recent Swedish survey of 753 students in their last year of teacher education found substantial variation in opportunities to learn specific competencies of mathematical teaching, such as analyzing learners’ answers or leading a mathematical discussion, as well as insufficient opportunities to learn from practical experience while in training (Christiansen and Erixon, 2021). Among Nordic countries, the Finnish system stands out in a positive way and might be an interesting model for Scandinavia (Tatto et al., 2012). Because MCK and MPCK seem to explain students’ mathematical gains, it could be that the need for acceleration is lower in classrooms where teachers score higher in both domains. Nevertheless, the general scores for Norwegian mathematical teachers can explain the need for acceleration overall. However, the quality of instruction in accelerated programs is connected to teachers’ mathematical content knowledge and mathematical pedagogical knowledge. Therefore, acceleration should only be considered sufficient if we can somehow guarantee that the instruction in those specific classrooms can meet the needs of mathematically gifted students. In Scandinavia, where we generally aim to meet all students’ needs in the ordinary inclusive classroom, we should focus on strengthening future teachers’ mathematical knowledge to ensure that they can meet all students’ needs. A lack of academic challenges seems to lead to boredom in school, which can have negative consequences. However, in this study, the students reacted differently to boredom. Thus, they seem to share an ability to cope with boredom over time and, therefore, have not become underachievers or developed other difficulties in school.

### Limitations and Future Studies

The students can only describe their own experience with their teachers’ knowledge. Therefore, we cannot know whether their experience represents a coherent picture of the level of knowledge among teachers who have educated them throughout school. In Norway, identification and recruitment of students to acceleration programs tend to be based on their performance (Smedsrud, 2018) and not through screening aimed at gifted students or to identify masked potential in the student population. In this way, we are truly dependent on teachers who have high knowledge in mathematics not only to foster mathematically gifted students in class but also to identify them for acceleration programs. Moreover, teacher training and knowledge of mathematically gifted students is essential for teachers to be able to identify them, especially students with masked potential (Al-Hroub and Whitebread, 2008). There might be a mismatch between teachers’ knowledge of giftedness and their responsibility to identify gifted students for such programs.

The interviews were limited by the interviewer’s ability to understand the participants’ answers and ask good follow-up questions that reflect the participants’ views. Furthermore, the selection criterion for this study was performance, which can present a limited view of giftedness because these students might also underachieve. It would have been interesting to gather intelligence scores to better validate the selection criterion. At the same time, a study by Smedsrud (2018) showed a high correlation between grades earned in mathematics and intelligence in Norwegian secondary schools. Underachieving gifted students might have a completely different experience with teachers than students who achieve at a high level. At the same time, teachers need competence to identify and foster mathematical creativity, even at lower levels of schooling. The latter might help teachers recognize the characteristics of mathematically creative students, even if the teachers do not have high mathematical competency. No single qualitative study can be generalized, especially in interview settings where open-ended questions are used. Different follow-up questions were asked of different participants based on their answers and focus. Replication of the interviews is not possible, and generalization of the findings to other contexts is limited.

## Data Availability Statement

The datasets presented in this article are not readily available because we need to delete any audio-file after 1 year due to Norwegian laws in regard to NSD. Requests to access the datasets should be directed to JS, jorgen.smedsrud@nifu.no.

## Ethics Statement

The studies involving human participants were reviewed and approved by Norwegian Centre for Research Data. Written informed consent to participate in this study was provided by the participants’ legal guardian/next of kin.

## Author Contributions

JS designed the study, gathered data, and conducted and analyzed the interviews in connection with the new categories presented in this manuscript. AN-H provided critical commenting during the different versions of the draft, contributed to the discussion of the themes in the article and the discussion section, and helped in developing and discussing the themes in the manuscript. EI contributed with critical comments, dialogue throughout the process, discussion of the analyses, and developing categories as well as developing the protocol for interviews. All authors contributed in writing and discussing the manuscript at several stages of the process.

## Conflict of Interest

The authors declare that the research was conducted in the absence of any commercial or financial relationships that could be construed as a potential conflict of interest.

## Publisher’s Note

All claims expressed in this article are solely those of the authors and do not necessarily represent those of their affiliated organizations, or those of the publisher, the editors and the reviewers. Any product that may be evaluated in this article, or claim that may be made by its manufacturer, is not guaranteed or endorsed by the publisher.

## Footnotes

1 In Norway, grades five and six present the most difficult math in senior high school and high school.

2 Competence level 3 refers to teachers who have high competence in mathematics and thereby can identify and foster creative students in mathematics (see Hoth et al., 2017).
